# Tumour volume as a predictor of postoperative speech impairment in children undergoing resection of posterior fossa tumours: a prospective, multicentre study

**DOI:** 10.1007/s00701-025-06459-x

**Published:** 2025-04-03

**Authors:** Aske Foldbjerg Laustsen, Shivaram Avula, Jonathan Grønbæk, Barry Pizer, Per Nyman, Pelle Nilsson, Radek Frič, Magnus Aasved Hjort, Vladimír Beneš, Peter Hauser, Beatrix Pálmafy, Giedre Rutkauskiene, Florian Wilhelmy, Rick Brandsma, Astrid Sehested, René Mathiasen, Marianne Juhler

**Affiliations:** 1 Department of Neurosurgery, Rigshospitalet, Copenhagen, Denmark; 2 Department of Paediatrics and Adolescent Medicine, Rigshospitalet, Copenhagen, Denmark; 3 Department of Radiology, Alder Hey Children’S NHS Foundation, Liverpool, UK; 4 University of Liverpool, Liverpool, L69 3BX UK; 5 Crown Princess Victoria Children’S Hospital and Department of Biomedical and Clinical Sciences, Linköping University, Linköping, Sweden; 6 Department of Medical Sciences/Neurosurgery, Uppsala University, Uppsala University Hospital, Uppsala, Sweden; 7 Department of Neurosurgery, University Hospital Oslo, Oslo, Norway; 8 Department of Pediatric Hematology and Oncology, St Olavs Hospital, Trondheim, Norway; 9 Department of Neurosurgery, 2nd Medical Faculty and Motol University Hospital, Prague, Czechia; 10 2nd Department of Pediatrics, Semmelweis University, Budapest, Hungary; 11 National Institute of Neuroscience, Budapest, Hungary; 12 Department of Pediatrics, Lithuanian University of Health Science, Kaunas, Lithuania; 13 Department of Neurosurgery, University Hospital Leipzig, Leipzig, Germany; 14 Princess Maxima Center for Pediatric Oncology, Utrecht, Netherlands

**Keywords:** Volumetry, Postoperative speech impairment, CMS, PFS, Posterior fossa tumour, Paediatrics

## Abstract

**Background:**

Cerebellar Mutism Syndrome (CMS) is a neurological complication of posterior fossa (PF) tumour surgery in children, and postoperative speech impairment (POSI) is the cardinal symptom of CMS. The role of tumour volume on the risk of POSI remains unexplored. This study investigates the association between tumour volume and the risk of POSI.

**Methods:**

We included 360 patients from the European CMS study with available preoperative T1-weighted contrast-enhanced brain MRI. Speech status was assessed within two weeks postoperatively and categorised into three levels: habitual speech, severely reduced speech, and mutism. Tumour volumes were calculated using the BrainLab Elements SmartBrush™, a semi-automated segmentation tool. We used proportional odds models to estimate the odds ratio (OR) with adjustments for tumour location, pathology, and age. Based on the primary analysis, a risk stratification model for medulloblastoma patients was constructed, and the optimal volume cut-off was determined with Youden’s Index.

**Results:**

We found no effect of the overall tumour volume on the risk of POSI. This result did not change when adjusted for tumour location, pathology, and age. We found an association between tumour volume of medulloblastoma and the risk of POSI (unadjusted OR of 1.04 per increase in cm^3^ (95% CI 1.01;1.07, *p* = *0.01*)), which did not change when adjusting for tumour location and age. The risk stratification cut-off for the tumour volume of medulloblastoma was calculated to be 16,5 cm^3^. Patients with medulloblastoma and preoperative tumour volumes below 16,5 cm^3^ had an absolute risk of 13% for POSI (low-risk group), whereas patients with preoperative tumour volumes above 16,5 cm^3^ had an absolute risk of 50% for POSI (high-risk group).

**Conclusion:**

Our data showed an association between preoperative tumour volume and the risk of POSI in children with medulloblastoma, while no association was found for the volume of other tumour types. We suggest a straightforward cut-off risk model for assessing the risk of POSI in children with medulloblastoma based on preoperative tumour volume. This approach can aid clinicians in informing patients and parents about the complications related to CMS following PF tumour surgery in children.

**Clinical Trials:**

ID NCT02300766 (October 2014).

**Supplementary information:**

The online version contains supplementary material available at 10.1007/s00701-025-06459-x.

## Introduction

Tumours of the central nervous system are the most common solid tumours in children, with an incidence of 2–3.5 cases per 100,000, and 55% arising in the posterior fossa (PF) [22]. Cerebellar mutism syndrome (CMS) is a severe complication of paediatric PF tumour surgery. The main symptom of CMS is postoperative speech impairment (POSI), seen in 30% of cases and usually transient, with POSI defined as either reduced speech limited to single words or short sentences, only elicited by direct prompting, or mutism [6]. Additional symptoms include emotional lability, hypotonia and ataxia [9]. The current understanding of the mechanisms involved in CMS consists of the disruption of cerebello-cerebral outflow tracts, namely the dentato-thalamo-cortical pathway (DTCp), leading to cerebello-cerebral diaschisis [2]. Although symptoms of CMS are generally temporary, studies have shown negative long-term implications for survivors of CMS, including movement disabilities, reduced neurocognitive performance, and persistent speech impairment [12, 25].

While the understanding of risk factors of CMS is advancing, the role of tumour size remains largely unexplored. Most studies focus on the lower patient age, midline tumour location, and tumour pathology of medulloblastoma (MB) as risk factors, with limited attention to tumour size [20]. Simplified proxies for tumour size, such as maximal tumour diameter [4, 21] and tumour area (calculated by multiplying the two largest perpendicular diameters) [16] have also been suggested as risk factors for CMS. Only one study has found an association between tumour volume – calculated as an ellipsoid approximation (ABC/2, A = largest diameter in the axial plane, B = largest perpendicular diameter to A in the axial plan, C = craniocaudal diameter in multiplanar reformat) [14] – and risk of CMS in a retrospective setting [27].

A study focusing on increased accuracy in 3D volume measurement could provide more precise insights into the correlation between tumour volumes and CMS risk. These insights may be crucial for improving risk stratification and guiding surgical approaches to mitigate the risk of POSI. Semi-automated segmentation for tumour volume has demonstrated high inter- and intrarater reliability in glioblastoma using the SmartBrush™ tool from BrainLab; this method is used in neurosurgical centres globally for preoperative tumour outlining and volume measurement, showing consistent and precise measurements across different users, including non-experts [10]. Its combined accuracy and general availability could thus be useful in this context.

Our study aims to investigate whether larger PF tumour volumes, calculated with a semi-automated segmentation model, are associated with a higher risk of POSI. This is based on the hypothesis that larger tumours may necessitate more extensive surgical manipulation as well as compress or infiltrate critical anatomical regions associated with POSI. We further hypothesise that 1) an association between the tumour volume and the risk of POSI will be stronger for high-graded tumour types, and 2) tumours infiltrating or compressing the brainstem or the 4th ventricle are more likely to result in POSI than similarly sized tumours located in the vermis or cerebellar hemisphere.

Addressing these hypotheses may improve risk assessment, surgical planning, and ultimately, patient outcomes in the management of paediatric PF tumours.

## Material and methods

### Study design

The "European study of the cerebellar mutism syndrome in children with brain tumours of the posterior fossa" (the CMS study) is a prospective, observational, multicentre cohort study focusing on the investigation of various risk factors for the development of CMS, including neuroradiographic biomarkers. Details of the CMS study design have been previously published [28].

### Participants

Children under 18 years of age who underwent resection or open biopsy for a PF tumour between 2014 and 2023 in 29 participating centres across 13 countries were prospectively included in this study. Informed consent was obtained before enrolment. The study design also permitted postoperative inclusion for emergency surgeries, allowing consent within seven days following surgery.

### Data collection

Basic information, including age and sex, was collected at inclusion. Patients were examined preoperatively and 1–4 weeks postoperatively with speech assessment and neurological examination. The operating neurosurgeon registered surgical details, including tumour location, within 72 h after surgery.

Tumour location was registered by the neurosurgeon into one or multiple of the five categories depending on its structural involvement: 1) brainstem, 2) 4th ventricle, 3) cerebellar vermis, 4) right cerebellar hemisphere, 5) left cerebellar hemisphere. The attending paediatrician or neurosurgeon documented the postoperative speech status as either 'yes' or 'no' for the presence of mutism. If the response was 'no,' an additional query was made regarding reduced speech, also recorded as 'yes' or 'no.' If both mutism and reduced speech were marked as 'no,' the speech was classified as habitual [6–8]. The data on tumour pathology were collected within two months of surgery and registered in 1 of 5 categories: 1) pilocytic/pilomyxoid astrocytoma, 2) medulloblastoma (MB), 3) ependymoma, 4) atypical teratoid/rhabdoid tumour (AT/RT), 5) other (Supplementary Table A). All data was registered in a secure online database.

### Tumour volumetry

Preoperative T1 contrast-enhanced Brain MRI was collected from routine clinical MRI. Scans were pseudonymised with DICOMReader PRO (v6.0.3) and imported to BRAINLAB Elements for “Cranial Planning”. The first author performed a semi-automated segmentation of the primary PF tumour outlining and tumour volumetry using SmartBrush™. SmartBrush™ is a region-growing algorithm that assists with 2D segmentation. The central part of the tumour is manually delineated and then interpolated into 3D using an additional 2D segmentation from a perpendicular slice. Manual adjustments to the segmentation can be made by adding or erasing specific regions of interest, either with the support of the region-growing algorithm or manually. All visible parts of the tumour were included in the volumetric calculations (cystic lesions, solid lesions, contrast-enhancing and non-contrast-enhancing lesions). In the case of multiple and anatomically isolated lesions, the larger lesion was considered the primary tumour, and the remaining lesions were considered metastases not included in the tumour volume.

### Variable stratification

POSI was stratified into three levels: 1) habitual speech, 2) reduced speech, and 3) mutism. Tumour location was divided into four mutually exclusive categories based on POSI risk stratification: 1) brainstem involvement, 2) 4th ventricle without brainstem involvement, 3) vermis without brainstem or 4th ventricle involvement, 4) cerebellar hemisphere without brain stem or 4th ventricle or vermis involvement).

### Statistical analyses

We evaluated POSI using a proportional odds model, investigating its association with tumour volume (cm^3^) as a linear variable. Adjustments were made for tumour pathology, location, and age, with results presented as proportional odds ratios (OR) and 95% confidence intervals (CI). No adjustments were made for multiple testing, and analyses were conducted on complete cases without imputation. Stepwise exclusions of patients with missing data for included confounders showed no impact on conclusions. Brant's test confirmed the proportional odds assumption in univariate and multivariate analyses.

Analyses on MB tumour volume were performed using an odds ratio model for POSI, unadjusted and adjusted for tumour location and age. A combination of logistic regression and Reciever Operating Characteristics (ROC) analysis was used to identify the optimal tumour volume cut-off associated with POSI. The speech variable was dichotomised into binary levels (0 = habitual, 1 or 2 = POSI), which aligned with results from the ordinal outcome (Supplementary Table B). Due to small subgroups in the ordinal outcome, binary grouping was deemed more appropriate. Logistic regression predicted POSI based on tumour volume, and ROC analysis determined the cut-off using Youden's Index [11]. The cut-off was adjusted to ensure clinical relevance and data fitting by setting a minimum threshold at the 20th percentile of tumour volume. This was done to reduce the impact of small tumour volumes not clinically linked to POSI, thereby avoiding overclassifying patients as "high risk." The approach ensured the cut-off reflected clinically significant tumour sizes with meaningful relevance to POSI risk. Patients were subsequently classified into high-risk and low-risk groups based on the adjusted cut-off. The risk classification was validated by comparing the absolute risks of POSI within these groups.

We conducted a sensitivity analysis on a subset of 57 randomly selected scans using the traditional ellipsoid volume approximation method (ABC/2). This analysis did not change our chosen method for calculating tumour volume, which was considered more appropriate (Supplementary Table C).

We performed a sensitivity analysis adjusting for individual country effects on POSI, which did not alter the overall conclusion (data not shown). Additionally, we tested a logarithmic transformation of tumour volume to address slight right skewing in volumetry. However, the transformation resulted in strong left skewing and did not improve model fit, leaving our statistical approach and overall conclusion unchanged (data not shown).

R-studio (v.2023.06.1) was used to perform statistical analyses.

## Results

Out of 725 included patients in the CMS study between 2014 and 2023, 360 patients had preoperative contrast-enhanced T1 brain MRI available and were included in this study. There were no considerable systematic differences between the included patients and the group without available preoperative contrast-enhanced T1 scan except for the extent of missing data (Table 1 and Supplementary Table D). The higher proportion of missing data in the non-included group likely reflected a pattern where the absence of available imaging was associated with incomplete data registration.

**Table 1:** Demographics of the included cohort

	All patients (*n* = 360); *N* (% of total)	Habitual speech (*n* = 253; 71%); *N* (%^c^)	Reduced speech (*n* = 60; 16%); *N* (%^c^)	Mutism (*n* = 47; 13%); *N* (%^c^)
Sex				
Male	203 (57)	138 (68)	38 (19)	27 (13)
Female	157 (43)	115 (73)	22 (14)	20 (13)
Age (Years; Median [IQR^a^])	7.2 [4.3;11.0]	8.0 [4.6;11.9]	6.7 [4.1;8.8]	4.8 [2.3;7.3]
Tumour volume (cm^3^; Mean (range))	30.4 (0.7;178.6)	32.4 (0.9;117.8)	31.5 (0.7;77.6)	32.0 (3.5;178.6)
Tumour pathology				
Pilocytic astrocytoma	157 (44)	131 (83)	16 (10)	10 (6)
Medulloblastoma	111 (31)	64 (58)	28 (25)	19 (17)
Ependymoma	37 (10)	23 (62)	5 (14)	9 (24)
AT/RT	11 (3)	3 (27)	4 (36)	4 (36)
Other	29 (8)	21 (72)	4 (14)	4 (14)
Unknown	15 (4)	11 (73)	3 (20)	1 (7)
Tumour location^b^				
Brain stem	67 (19)	40 (60)	14 (21)	13 (19)
4th ventricle	120 (33)	66 (55)	25 (21)	29 (24)
Vermis	72 (20)	57 (79)	12 (17)	3 (4)
Cerebellar hemisphere	93 (26)	84 (90)	8 (9)	1 (1)
Unknown	8 (2)	6 (75)	1 (13)	1 (13)

^a^Interquartile range; ^b^Brainstem involvement, 4th ventricle without brainstem involvement, vermis without brainstem or 4th ventricle involvement, cerebellar hemisphere without brain stem or 4th ventricle or vermis involvement; ^c^% of total in category (horizontal))

**Fig. 1:**
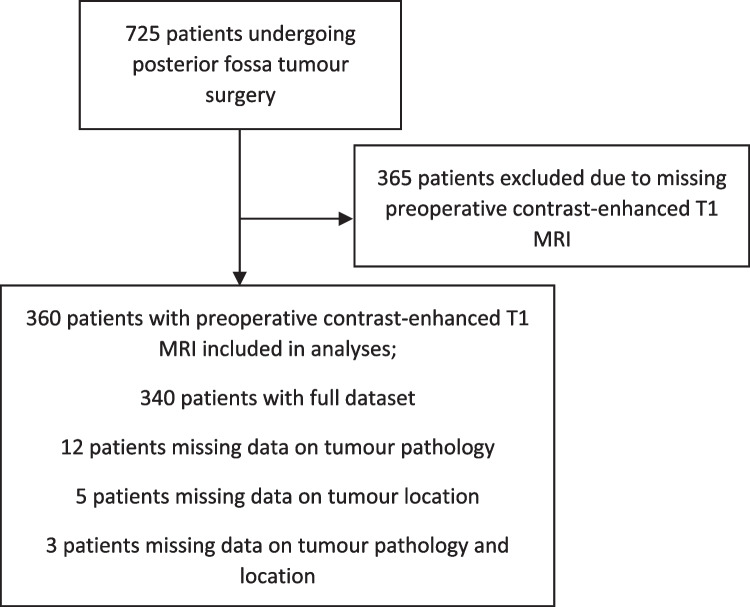
Patient flow chart

Out of the 360 patients, 15 patients (4%) had missing tumour pathology, and eight patients (2%) had missing tumour location (three patients missed information on both tumour pathology and location), leaving 340 patients with full dataset (Fig. 1). Demographic data of the patient population are summarised in Table 1. 204 (57%) were male. Median age was 7.2 (IQR 4.3;11.0). The mean tumour volume was 30.4 cm^3^, ranging from 0.7 to 178.6 cm^3^. Predominant tumour pathology was pilocytic astrocytoma (*n* = 157; 44%), followed by MB (*n* = 111; 31%), ependymoma (*n* = 37; 10%), other (*n* = 29; 8%) and AT/RT (*n* = 11; 3%). The predominant tumour location, based on the exclusive categories of structural involvement, was the 4th ventricle (*n* = 120; 33%), followed by the cerebellar hemispheres (*n* = 93; 26%), the vermis (*n* = 72; 20%) and brainstem involvement (*n* = 67; 19%). POSI was present in 107/360 (29% (reduced speech: *n* = 60; 16%; mutism: *n* = 47; 13%)).

### Primary analysis

Results from the univariate and multivariate proportional odds models are presented in Table 2. No significant association was found between the tumour volume and the risk of POSI (unadjusted OR 1.00, 95% CI 0.97–1.01). Adjustments for tumour pathology, location, and age did not alter this finding (adjusted OR 0.99, 95% CI 0.97–1.01). A statistically significant interaction between the pathology of MB and tumour volume on the risk of POSI was found, justifying the decision to conduct subanalyses focusing on MB pathology (Supplementary Table E).

**Table 2:** Risk of postoperative speech impairment

Univariate analysis		Multivariate analysis		
	(*n* = 360)	Model 1 (adjustments: tumour pathology, *n* = 345)	Model 2 (adjustments: Model 1 + tumour location, *n* = 340)	Model 3 (adjustments: Model 2 + age, *n* = 340)
Tumour volume (OR per 1 cm^3^ increase**)**	1.00 (0.97;1.01) *p* = *0.57*	0.99 (0.97;1.01) *p* = *0.20*	0.99 (0.97;1.01) *p* = *0.35*	0.99 (0.97; 1.01) *p* = *0.29*

### Subanalyses for medulloblastoma

Our results indicated a 4% increase in POSI risk for each cubic centimetre increase in MB tumour volume (unadjusted OR 1.04, 95% CI 1.01;1.07). Multivariate adjustments did not change this result (adjusted OR 1.04, 95% CI 1.01;1.08, *p* = *0.01*). Subanalyses for MB patients are presented in Table 3.

**Table 3:** Risk of postoperative speech impairment (binary outcome) in medulloblastoma

Univariate analysis		Multivariate analysis	
	(*n* = 111)	Model 1 (adjustments: tumour location, *n* = 110)	Model 2 (adjustments: Model 1 + age, *n* = 110)
Tumour volume (OR per 1 cm^3^ increase)	1.04 (1.01;1.07) *p* = *0.01*	1.04 (1.01;1.08) *p* = *0.009*	1.04 (1.01;1.08) *p* = *0.01*

**Fig. 2:**
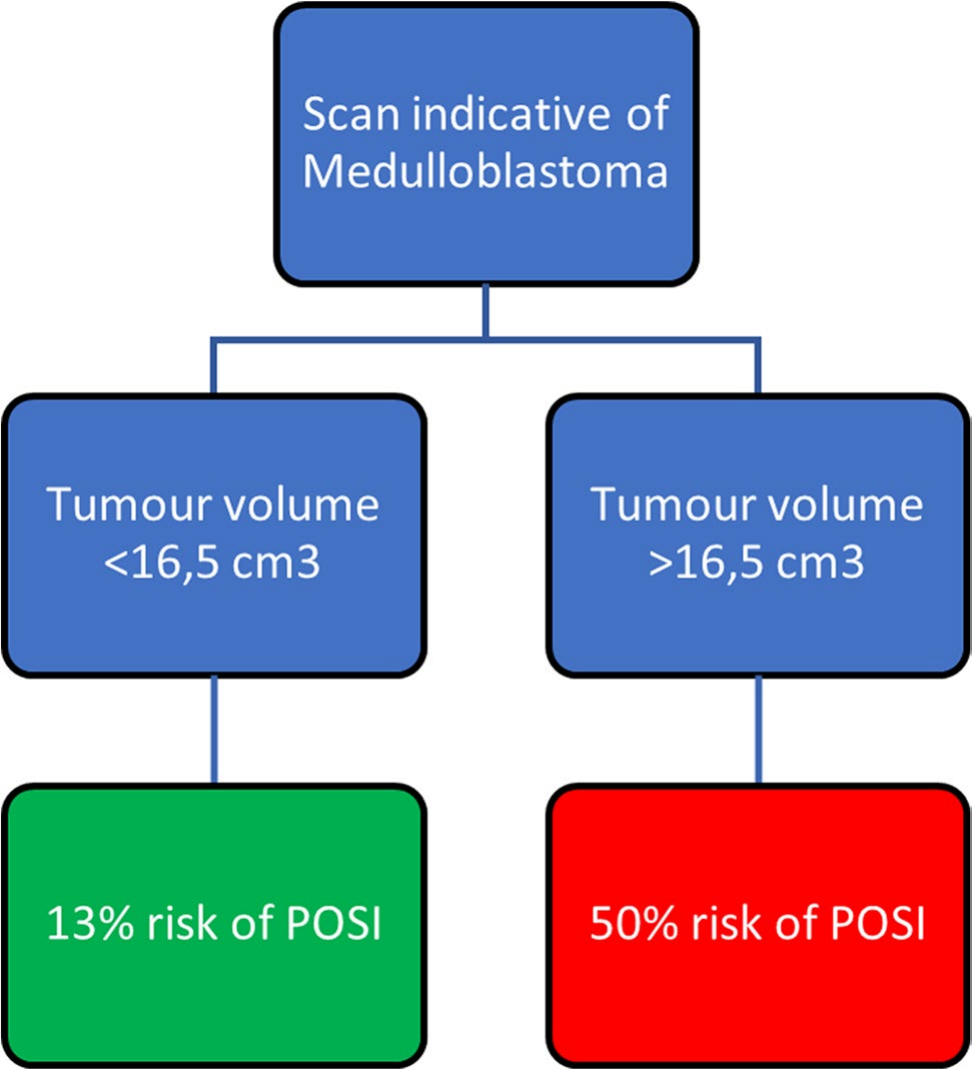
Absolute risk prediction model for medulloblastoma

We identified an optimal tumour volume cut-off for risk stratification at 16.5 cm^3^ (Supplementary Tables F and G, Supplementary Figs. 1 and 2). This resulted in an absolute risk of 13% for POSI when MB tumour volume was less than 16.5 cm^3^ and 50% when more than 16.5 cm^3^, respectively (Table 4, Fig. 2).

**Table 4:** Risk classification for medulloblastoma

	Habitual	POSI	Absolute risk of POSI
Low risk (Tumour volume < 16.5 cm^3^)	20	3	13%
High risk (Tumour volume > 16.5 cm^3^)	44	44	50%

## Discussion

### Tumour volumetry and risk of POSI

We found an association between tumour volume in children with MB and the risk of POSI. We did not find such an association for the remaining tumour types; therefore, there was no overall effect of tumour volume. This can be explained by the study design, which included all PF tumours undergoing open surgery. The most frequent was pilocytic astrocytoma, where the risk of POSI is generally considered lower than in more malignant PF tumours like MB, which was confirmed by present findings. Furthermore, the extent of peritumoral oedema may contribute to the risk of POSI. This potentially occurs by complicating surgical challenges at the tumour-brain border or increasing pressure on surrounding structures, heightening their vulnerability to surgical damage.

Another possible explanation, based on previously suggested hypotheses [1], could be thermal or mechanical perioperative injury, as tumours with higher grades of malignancy often require more radical resection strategies. This increases the likelihood of damaging critical PF structures, including proximal efferent cerebellar pathways and the fastigial nucleus, thus increasing the risk of postoperative complications such as POSI [27]. The use of an ultrasonic aspirator at the tumour-brain border has been proposed to cause this type of thermal injury [1]. Firmer tumours, especially, may require more aggressive settings of the ultrasonic aspirator, increasing the likelihood of damage to surrounding brain tissue. However, based on two publications [23, 27], a recent review found no overall association between the use of ultrasonic aspirator and the incidence of CMS [20]. Additionally, surgical manipulation of the tumour tissue near critical pathways can increase the risk of mechanical injury, regardless of the tumour's size. A more detailed stratification of tumour location and peroperative structural manipulation than applied in the present study may be necessary to fully appreciate the impact of tumour size on the risk of POSI.

It is important to note that this study found no association between the risk of POSI and tumour volume in other high-grade tumours (ependymoma or AT/RT). Several factors may explain this lack of association: 1) the number of patients in both groups was relatively modest, resulting in uncertainty of our results specifically for these tumour subtypes; 2) ependymomas tend to have a “plastic” growth pattern somewhat respecting the anatomy of the 4th ventricle and surrounding structures, without necessarily compressing or invading these structures; and 3) AT/RT typically occurs in very young patients, where speech assessment is inherently challenging, and age itself acts as a strong independent risk factor for POSI [6].

### Risk stratification of POSI based on medulloblastoma tumour volume

By stratifying the subgroup of patients with MB into “low” and “high” risk groups based on the tumour volume, our results can potentially guide neurosurgeons when informing children and their parents about the risk of POSI in a clinical setting. Furthermore, our results may aid in surgical planning, as the volumetric calculation method applied in our study is straightforward and widely used in neurosurgical practice [10]. This method may also be beneficial by increasing the surgeon's awareness of whether the tumour is adjacent to or invading critical structures related to CMS when outlining the tumour during preoperative planning. A key surgical challenge in MB resection is distinguishing tumour tissue from adjacent deep cerebellar and brainstem nuclei, as well as the cerebellar outflow tracts, particularly in large midline tumours, which thin, abut or invade these structures. Avoiding damage to the deep cerebellar nuclei and DTCp may be more critical in reducing the risk of POSI than considering the tumour size alone. In particular, the superior cerebellar peduncles and dentate nuclei are considered critical structures, as unilateral or bilateral damage has been suggested to cause CMS by disruption of the cerebello-cerebral outflow tracts [16–19, 24]. However, assessing this factor would require the integration of advanced imaging techniques that were not available in our cohort.

### Semi-automated tumour volume segmentation

We chose to apply a different approach to tumour volumetrics than the traditional ellipsoid approximation calculation (ABC/2) because we believe that the method used in our study is more representative of the actual tumour size. We did not find any association between the ABC/2 overall tumour volume estimation and the risk of POSI. The ABC/2 method tends to overestimate the actual tumour volume, particularly in cases where the tumour shape deviates from an ellipsoid or has more heterogeneous geometrical characteristics. This method was originally intended to estimate the volume of a haemorrhagic lesion in acute settings but has since gained traction in other types of brain lesions, including tumours [14, 15]. The inaccuracy is also evident in our results (ABC/2 mean volume 61.64 cm^3^ vs semiautomated segmentation mean volume 30.4 cm^3^), making it challenging to draw associations between the tumour volume estimation from ABC/2 and the risk of POSI. By outlining the tumour with semi-automated segmentation, a more precise tumour volume can be calculated, providing a better fit to the actual volume of the tumour [13]. Furthermore, measuring diameter in three planes does not outline critical locations of the tumour and is, therefore, of less clinical relevance to the neurosurgeon during preoperative planning.

### Limitations

#### Choice of imaging

Our choice of method comes with certain limitations. Tumour volumetrics were performed on T1-weighted contrast-enhanced imaging, essential for neurosurgical planning and the most consistently available sequence across centres. For non-contrast-enhancing tumours, distinguishing normal from pathological tissue relied on anatomical knowledge and anatomical deviations suggestive of tumour pathology, introducing some uncertainty in the semi-automated segmentation process. Tumour borders might have been better delineated with sequences like T2, but data availability restricted our analysis to T1-contrast-enhanced imaging. Multicentre data transfer posed challenges in this context, with large file sizes often leading to missing sequences or low-quality scans, preventing access to sequences like T2, T2 FLAIR, or DWI. While specific neuro-radiological methods for anatomical segmentation of PF pathology exist, potentially offering more precise volumetrics [13], we selected a commercially available and widely used software to ensure accessibility in neurosurgical practice, thereby underscoring the clinical applicability of our results. Future studies incorporating additional MRI sequences or employing a more comprehensive volumetric segmentation method could help validate our findings.

#### Challenges with subjectivity

The inherent subjectivity in tumour volume assessment is a critical factor contributing to variability in measurements, with experience being the most influential determinant. A prior study on glioblastoma tumour outlining demonstrated high interobserver agreement using this volumetric method [10]. While not validated in a paediatric neuro-oncological setting, this method is widely recognised as a clinical tool in neurosurgical planning. Additionally, the subjectivity in speech assessment must be acknowledged, as it introduces variability in evaluating postoperative speech deficits.

#### Preoperative assessment of tumour typology

Our risk stratification model relies on the histopathological diagnosis of MB, whereas preoperative risk assessment must be based on radiological criteria, which may impact its clinical application. However, a previous study reported high sensitivity between radiological and pathological diagnoses for paediatric PF tumours [5]. While MB has distinct neuroradiographic features, such as increased cellularity on CT, specific T2 signal characteristics, and DWI patterns, other tumours, like ependymoma, can occasionally mimic its appearance, potentially misleading even experienced paediatric neuroradiologists. In such cases, neurosurgeons may overestimate the risk of POSI based on our model. This highlights the importance of diagnostic accuracy, as the reliability of predictive risk stratification models generally depends on accurate radiological tumour diagnosis [3, 26].

#### Postoperative imaging correlates

While this study focused on the clinical outcome of POSI, we acknowledge the growing body of evidence linking CMS to postoperative disruptions in the Guillain-Mollaret triangle, including tract disruption and hypertrophic olivary degeneration [2, 29]. Incorporating postoperative imaging findings could improve the validity of our outcome measure. However, a qualitative analysis of postoperative imaging has not yet been conducted. This remains an important focus for future research. We are currently conducting integrated longitudinal MRI assessments on our cohort to further explore the structural correlates of CMS.

#### Missing data

A multicentre or multinational setting often provides challenges in data completeness, which is evident in this study, with only 360/725 patients with preoperative contrast-enhanced T1 MRI sequences collected centrally. Consequently, this may have introduced a risk of selection bias. Fortunately, no systematic differences were observed between the cohort of patients included in this study and the overall cohort in the CMS study. It can thus be safely assumed that the study’s findings are representative of the entire CMS study cohort.

### Strengths

The main strengths of our study are the number of patients included and its prospective design, ensuring robustness of our results while minimising possible biases. Although our results need to be validated in independent cohorts, the multicentre setting provides some degree of external validity to our results. Studies focusing specifically on ependymoma or AT/RT are required to assess the impact of their tumour volume on the risk of POSI and other CMS symptoms. Furthermore, future research should explore additional factors that may influence the risk of POSI, including genetics.

## Conclusion

Our data showed an association between preoperative tumour volume and the risk of POSI in children with medulloblastoma, while no association was found for the volume of other tumour types. We suggest a straightforward cut-off risk model for assessing the risk of POSI in children with medulloblastoma based on preoperative tumour volume. This approach can aid clinicians in informing patients and parents about the complications related to CMS following PF tumour surgery in children.

## Supplementary Information

Below is the link to the electronic supplementary material.

ESM 1 (PDF 267 KB)

## Data Availability

The data supporting the findings of this study are derived from the ongoing European CMS study, which is currently in the recruitment phase. The dataset is not publicly available due to the confidential nature of patient data and the need to protect study participant privacy. Anonymized data can be made available upon reasonable request to the corresponding author, pending completion of recruitment and data consolidation, and in compliance with ethical and institutional guidelines.

## Abbreviations

PF: Posterior fossa
CMS: Cerebellar mutism syndrome
POSI: Postoperative speech impairment
MB: Medulloblastoma
ABC: A = largest diameter in the axial plane, B = largest perpendicular diameter to A in the axial plan, C = craniocaudal diameter in multiplanar reformat
The CMS study: The European study of the cerebellar mutism syndrome in children with brain tumours of the posterior fossa
AT/RT: Atypical teratoid/rhabdoid tumour
OR: Odds ratio
CI: Confidence interval
ROC: Receiver operating characteristics
