# Supplementary material for: Tumour volume as a predictor of postoperative speech impairment in children undergoing resection of posterior fossa tumours: a prospective, multicentre study
Source: Acta Neurochir (Wien). 2025 Apr 3;167(1):97. doi: 10.1007/s00701-025-06459-x (PMC11968465; doi:10.1007/s00701-025-06459-x)
Supplement: Supplementary file 1 — (PDF 267 KB) [file 701_2025_6459_MOESM1_ESM.pdf]

Title

Tumour volume as a predictor of postoperative speech impairment in children undergoing resection of posterior fossa tumours: a prospective, multicentre study.

Journal: Acta Neurochirurgica

Authors

Aske Foldbjerg Laustsen[1,2], Shivaram Avula, Jonathan Grønbæk, Barry Pizer, Per Nyman, Pelle Nilsson, Radek Frič, Magnus Aasved Hjort, Vladimír Beneš, Peter Hauser, Beatrix Pálmafy, Giedre Rutkauskiene, Florian Wilhelmy, Rick Brandsma, Astrid Sehested, René Mathiasen, Marianne Juhler

Corresponding author: Aske Foldbjerg Laustsen (e-mail: aske.foldbjerg.laustsen@regionh.dk)

Affiliation of corresponding author:

[1]Department of Neurosurgery, Rigshospitalet, Denmark

[2]Department of Paediatrics and Adolescent Medicine, Rigshospitalet, Denmark

Supplementary material

Supplementary table A other tumours (as registered by the paediatrician or neurosurgeon)

Tumour classification included under ”other”-category	Amount (n = 29)
Diffuse midline glioma	5
Ganglioglioma	5
Glioblastoma	4
Haemangioblastoma	2
Schwannoma	2
Embryonal tumour with multilayered rosettes	1
Choroid plexus papilloma	1
Pineoblastoma	1
Meningioma	1
Mixed glioneuronal tumour	1
Diffuse low grade glioma without further specification	1
Astroglioma without further specification	1
Germ cell tumour without further specification	1
Subclassification not available	3

Supplementary table B ordinal speech outcome for medulloblastoma patients

Risk of postoperative speech impairment	Univariate analysis	Multivariate analysis 1	Multivariate analysis 2
**Tumour volume (**per 1 cm^3 increase**)**	1.03 (1.01; 1.06)	1.04 (1.01; 1.07)	1.04 (1.01; 1.07)
**Tumour location** (reference = 4th ventricle)			
Brain stem	-	1.39 (0.41; 4.74)	1.40 (0.41; 4.79)
Vermis	-	0.19 (0.06; 0.62)	0.19 (0.06; 0.62)
Cerebellar hemisphere	-	0.08 (0.02; 0.42)	0.08 (0.02; 0.42)
Age (per increase in year)	-	-	0.98 (0.88; 1.10)

Supplementary table C Tumour volume ABC/2

**Risk of postoperative speech impairment (ordinary, 3 levels)**	**Univariate analysis**	**Multivariate analysis 1 (Tumour type)**	**Multivariate analysis 2 (M1 + tumour location)**	**Multivariate analysis 3 (M2 + age)**
Tumour volume (ABC/2; OR per 1 cm^3 increase)<br>Number of observations: 57<br>Mean: 61.64<br>Range: 22.80;91.30	0.98 (0.94; 1.03)	0.98 (0.94; 1.03)	0.99 (0.94; 1.04)	0.98 (0.93; 1.04)

Supplementary Table D Demographics of excluded cohort (no available preoperative MRI T1 contrast-enhanced scan)

	All patients (n=365)		
	N	% of total	% of known status
**Sex**			
Male	205	56	58
Female	148	41	42
Unknown	12	3	
**Age** (Years; Median and Range)	6.8 (0.4; 17.8)		
**Tumour histology**			
Pilocytic astrocytoma	116	32	46
Medulloblastoma	74	20	29
Ependymoma	30	8	12
Atypical Rhabdoid/Teratoid tumour	3	1	1
Other	30	8	12
Unknown	112	31	
**Tumour location**			
Brain stem	76	21	24
4th ventricle	110	30	34
Vermis	61	17	19
Cerebellar hemisphere	75	21	23
Unknown	43	12	
**Postoperative speech**			
Habitual	228	63	78
Reduced	28	8	10
Mutism	38	10	12
Unknown	71	19	

Supplementary table E Risk of Postoperative Speech Impairment

Univariate analysis			Multivariate analysis		
			Model 1 (n=345)	*Model 2 (n=340)*	*Model 3 (n=340)*
	(*n=360*)	*Missing tumour pathology removed (n=345)*			
**Tumour volume (**per 1 cm^3 increase**)**	1.00 (0.97;1.01)	1.00 (0.97;1.01)	0.99 (0.97;1.01)	0.99 (0.97;1.01)	0.99 (0.97; 1.01)
**Tumour pathology**					
Pilocytic astrocytoma (PA)	-		1 (ref)	1 (ref)	1 (ref)
Medulloblastoma (MB)	-		0.95 (0.32;2.84)	0.63 (0.19; 2.04)	0.62 (0.19; 2.06)
Ependymoma (EP)	-		3.53 (0.72;17.37)	2.49 (0.45; 13.77)	2.15 (0.39; 11.82)
Atypical Teratoid/Rhabdoid Tumour (AT/RT)	-		3.21 (0.43;24.01)	3.88 (0.29; 52.14)	2.63 (0.18; 37.85)
Other	-		0.81 (0.22;3.00)	0.56 (0.14; 2.19)	0.69 (0.17; 2.78)
**Interaction between tumour volume and pathology**					
Tumour volume and PA	-		1 (ref)	1 (ref)	1 (ref)
Tumour volume and MB	-		1.04 (1.01;1.08)	1.04 (1.01; 1.08)	1.04 (1.01; 1.08)
Tumour volume and ependymoma	-		0.99 (0.94;1.05)	0.98 (0.92; 1.04)	0.97 (0.92; 1.03)
Tumour volume and AT/RT	-		1.01 (0.92;1.11)	1.01 (0.92; 1.12)	1.01 (0.91; 1.11)
Tumour volume and other tumours	-		1.03 (1.00;1.06)	1.03 (1.00; 1.06)	1.03 (0.99; 1.06)
**Tumour location**					
Brain stem	-		-	0.92 (0.47; 1.80)	0.88 (0.45; 1.72)
4th ventricle	-		-	1 (ref)	1 (ref)
Vermis	-		-	0.33 (0.16; 0.68)	0.32 (0.15; 0.66)
Cerebellar hemisphere	-		-	0.16 (0.07; 0.35)	0.17 (0.07; 0.39)
**Age (**per 1 year increase**)**			-	-	0.91 (0.85; 0.98)

Supplementary table F Odds ratio for POSI (yes/no) for medulloblastoma

Risk of speech impairment (binary outcome)	Univariate OR (Lower CI; Upper CI)	Multivariate analysis 1	Multivariate analysis 2
**Tumour volume (**per 1 cm^3 increase**)**	1.04 (1.01; 1.07)	1.04 (1.01; 1.08)	1.04 (1.01; 1.08)
**Tumour location** (reference = 4th ventricle)			
Brain stem	-	1.27 (0.32; 5.10)	1.28 (0.32; 5.13)
Vermis	-	0.22 (0.07; 0.74)	0.22 (0.07; 0.75)
Cerebellar hemisphere	-	0.09 (0.02; 0.45)	0.09 (0.02; 0.45)
Age (change per increase in year)	-	-	0.99 (0.88; 1.11)

Supplementary fig. 1 ROC curve

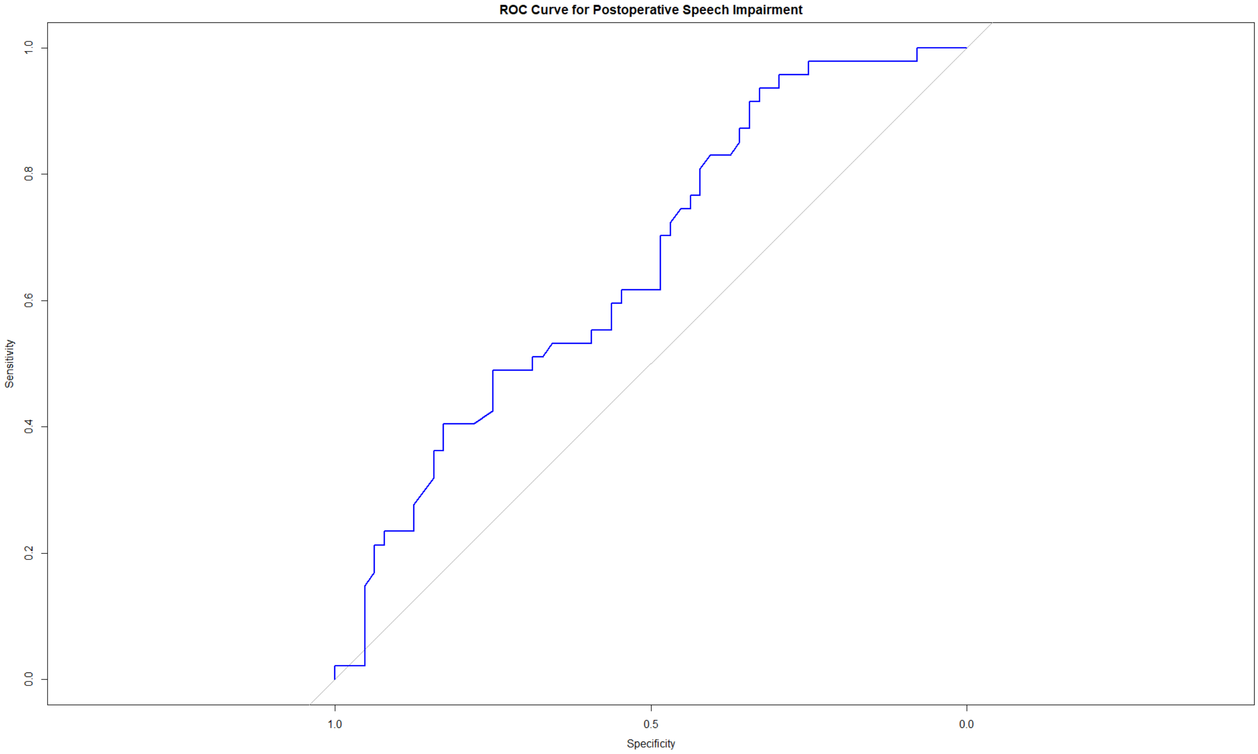

Supplementary fig. 2 Minimum threshold for medulloblastoma tumour volume in the risk prediction model

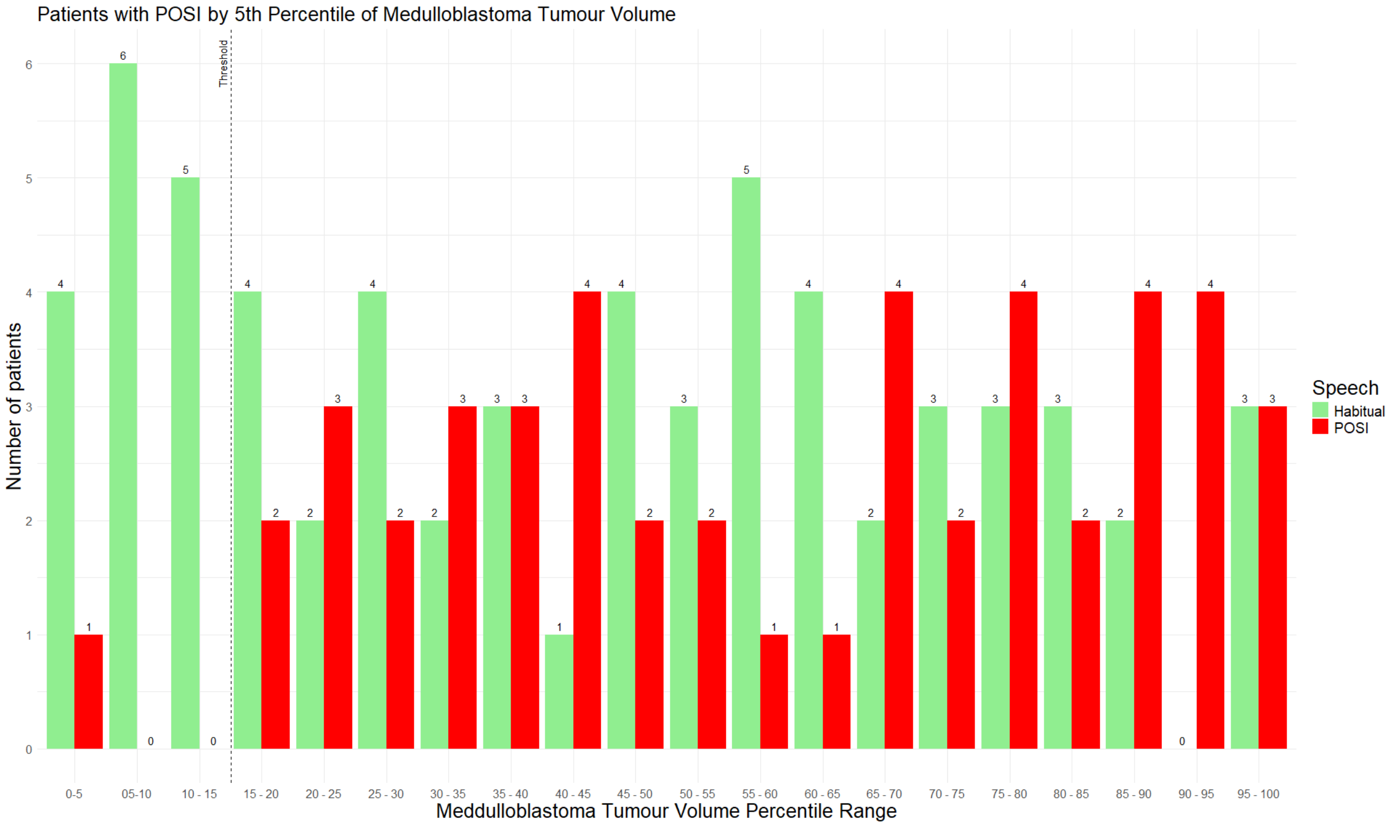

Supplementary table G Optimal volume cut-off

**Youden's index results**	**Predicted probability cut-off**	**Adjusted Optimal volume cut-off**
	0.34	16.5 cm^3
